# Behçet's disease

**DOI:** 10.1186/1750-1172-7-20

**Published:** 2012-04-12

**Authors:** David Saadoun, Bertrand Wechsler

**Affiliations:** 1 Service de Médecine Interne 2, Centre de Référence Maladies Autoimmunes rares, CHU Pitié-Salpêtrière, 83 Bd de l'Hôpital, 75013, Paris, France

**Keywords:** Behçet's disease, Uveitis, Vasculitis, Autoimmunity

## Abstract

**Definition of the disease:**

Behçet disease (BD) is a chronic, relapsing, multisystemic disorder characterized by mucocutaneous, ocular, vascular and central nervous system manifestations.

**Epidemiology:**

BD seems to cluster along the ancient Silk Road, which extends from eastern Asia to the Mediterranean basin. European cases are often described, not exclusively in the migrant population.

**Clinical description:**

The clinical spectrum includes oral and genital ulcerations, uveitis, vascular, neurological, articular, renal and gastrointestinal manifestations.

**Etiology:**

The etiopathogenesis of the disease remains unknown, although genetic predisposition, environmental factors and immunological abnormalities have been implicated.

**Diagnostic methods:**

Diagnosis is only based on clinical criteria.

**Differrential diagnosis:**

It depends **on** the clinical presentation of BD, but sarcoidosis, multiple sclerosis, Crohn’s disease, Takayasu’s arteritis, polychondritis or antiphospholipid syndrome **need to be considered**.

**Management:**

Treatment is symptomatic using steroids and immunomodulatory therapy. It is efficient depending on the rapidity of initiation, the compliance, and the duration of therapy.

**Prognosis:**

The prognosis is severe due to the ocular, neurological and arterial involvement.

## 

During the annual meeting of the Medical Association of Athens on November 15, 1930, Benediktos Adamantiades (1875–1962), Greek ophthalmologist from Prussa (nowadays Bursa, Turkey), presented in a lecture with the title "A case of relapsing iritis with hypopyon", a 20-y-old male patient with the three cardinal signs of the disease [1]. Adamantiades brought the genital ulcers, the arthritis, and the ocular signs in connection as signs of a single disease [1]. In 1937 Hulusi Behcet described three patients with oral and genital ulcerations and hypopyon uveitis. Since then, due to involvement of central nervous system, large vessels (veins and/or arteries), heart and rarely gastrointestinal tract or kidney, Behçet’s disease (BD) is considered as a widespread vasculitis. Behçet’s disease (BD) is a chronic systemic inflammatory disorder at the crossroad between autoimmune and autoinflammatory syndromes [2].

International criteria of classification have been defined with a sensitivity of 85% and specificity of 96% [3] (Table 1).

**Table 1:** International Classification Criteria Of Behcet's Disease

**In the absence of other clinical explanations, patients must have:**	
1. Recurrent oral ulceration (aphthous or herpetiform) observed by the physician or patient recurring at least three times in a 12-month period;	
**And two of the following:**	
2. Recurrent genital ulceration.	
3. Eye lesions: anterior uveitis, posterior uveitis, cells in the vitreous by slit lamp examination or retinal vasculitis observed by an ophthalmologist.	
4. Skin lesions: erythema nodosum, pseudofolliculitis, papulopustular lesions or acneiform nodules in postadolescent patients not on corticosteroids.	
5. Pathergy, read by a physician at 24–48 hours.	
(Sensibility 85% - Specificity 96%)	

## Epidemiology

Cases of BD seem to cluster along the ancient Silk Road, which extends from eastern Asia to the Mediterranean basin. The prevalence is 80 to 370 cases per 100,000 population in Turkey, 10/100,000 in Japan and 0.6/100,000 in Yorkshire. European cases are more often described, not exclusively in the migrant population. In common with ankylosing spondylitis and psoriatic arthropathy, BD has MHC class I associations. HLA-B51 is the most strongly associated known genetic factor to BD [4]. However, it accounts for less than 20% of the genetic risk, even in familial cases (less than 5%), which indicates that other genetic factors remain to be discovered. An association between the BD susceptibility allele HLAB51 with ocular disease has been reported [5]. A genetic contribution to BD is also supported by the high sibling recurrence risk ratio, estimated to be between 11.4 and 52.5 in the Turkish population [6]. The male to female ratio is 7:1 in symptomatic forms, but women predominate over men in studies were less symptomatic forms are systematically looked for and included [7]. BD **occurs** mainly between 18 to 40 years. Some paediatric onset cases are reported [8]. After 55 years, onset of BD is exceptional and diagnosis has to be made very cautiously.

## Clinical features

### Mucocutaneous lesions

The mucocutaneous lesions constitute the hallmark of BD.

Oral aphthae occur in 98% of cases and are mandatory in the international criteria of classification. Painful oral ulcers appear in the tongue, pharynx, buccal and labial mucosal membranes. They are unique or multiple. The typical lesion is round with a sharp, erythematous and elevated border, mostly 1 to 3 cm in diameter, but larger lesions can also occur. They can start as a raised redness and soon ulcerate. The surface is covered with a yellowish pseudomembrane. The lesions heal within about 10 days mostly without scarring. Like in all oral aphthoses, oral ulcers in BD can occur after physical or toxic influence (dental procedures, simple trauma, or certain foods such as nuts). When they are numerous or large, eating and/or speaking it is painful. They evolved to healing without scarring and/or lymph node swelling. They can not be differentiated from recurrent oral ulcers but their number, their size, their recurrence urge to look for the other manifestations of BD.

Genital aphthae occur in 60 to 65% of cases and are very suggesting of the diagnosis of BD. They are localized in men on the scrotum, less frequently on the penis or in the urethra and in women on the vulva and vagina where they can be disseminated and painful or totally indolent. They are morphologically similar to the oral ulcers but usually larger and deeper. The lesions leave scars in 50% of cases allowing retrospective diagnosis.

The ulcers can also occur on the esophagus, stomach, small intestine and perianal area, where they can lead to perforation.

The most frequent skin manifestations are pseudofolliculitis and erythema nodosum-like lesions. In contrast to acne vulgaris, pseudofolliculitis and acneiform nodules can appear all over the body and they are not always hair follicle-associated. Biopsy is useful to demonstrate an inflammatory infiltrate which is not connected with hair follicle. In our experience, the diagnosis is not possible when the patient is treated with steroids. A specific cutaneous irritability is frequent with papules on site of injections. The pathergy test, a 2mm papule induced 24 to 48 hours after a cutaneous pritch, is one of the 4 criteria of classification, but its sensivity is low with disposable needle and cutaneous disinfection.

## Eye manifestations

Ocular involvement is frequent and severe, often bilateral, compromising rapidly the visual function [9,10]. A variety of eye lesions have been found including anterior uveitis, cataract, glaucoma, posterior segment involvement with vasculitis, vitritis, retinitis, panuveitis, retinal edema, cystoid macular degeneration, venous or arterial occlusion, disc edema and retinal detachment.

Anterior uveitis with hypopyon despite first described is rare, transient and has a relative good prognosis. It can be complicated with synechia and glaucoma.

Posterior uveitis is almost constant in case of ocular involvement. Choroid is first involved with necrosis lesions. The lesions are peripheral and can lead to pigmentation. Vitreous body is involved secondarily with loss of transparency, retraction and traction on the retina. Fluorescein angiography show capillary dilatation, vascular obstruction and area of leak. Vasculitis is the basic process involving veins and arteries with tendency to thrombosis.

Prognosis is severe due to frequent relapses and partial recovery with therapy. The poor long-term visual prognosis is related to ocular hypertension, cataract and retinal vasculitis with blindness occurring in 25 to 30% of cases in most series at 5 years. In our experience, with intensive therapy and careful care simultaneously realized by ophthalmologist and internist, the prognosis is much better with only 2% of de novo severe visual loss at 6 years of follow-up.

The eye involvement may also be associated with neurological manifestations: cranial nerves palsy, optic neuropathy or papilledema with benign intracranial hypertension.

## Vascular manifestations

**Venous thrombosis** occurs in 30% of cases. BD is included in the wide spectrum of vasculitis. Vasculitis is a principal pathologic finding in BD, and vessels of all sizes are involved, both in the arterial and venous systems.

Venous disease is more common than arterial involvement, and its prevalence may account for 14% to 39% of patients with BD according to studies. Venous thrombosis in BD is a severe disorder, which may affect many different sites including the inferior vena cava, superior vena cava, pulmonary artery, supra-hepatic vessels and cardiac cavities.

*Superficial thrombophlebitis* are transient and migrant and sometimes misdiagnosed as erythema nodosum.

*Deep vein thrombosis* can be seen on various sites but especially on big vessels: iliofemoral, superior or inferior vena cava or on unusual localization such as dural sinus thrombosis (headache, papilledema, intracranial hypertension), hepatic veins (Budd-Chiari syndrome) or inferior vena cava with pulmonary aneurysms (Hughes-Stovin syndrome) [11].

These thrombophlebitis relapses frequently and must lead to the diagnosis in a young patient without any other thrombophilic factor. They occur generally in the first year after onset. Pulmonary embolism is not frequent, probably due to the inflammatory process on the endothelial wall of the vessel. In few cases, coincidental thrombophilic factors have been encountered.

**The arterial involvement** is seen in 3 to 5% of cares [12]. The incidence is probably underestimated because an autopsy survey showed that 33% of patients had arterial lesions, most of them had been asymptomatic [11,13]. Thrombosis and/or aneurysms are observed, mainly false aneurysms [14]. These "arterial aphthae" are localized on pulmonary arteries, aorta, renal and peripheral arteries. They can rupture suddenly. Vascular surgery is mandatory, but thrombosis of the graft and relapses of aneurysms at the site of bypass are frequent.

The pulmonary aneurysms have a severe prognosis, surgery and/or arterial embolization must be associated with corticosteroids and immunosuppressive drugs.

**Cardiac involvement** is also observed and may involve the 3 tunics [15,16]. Myocarditis, endocarditis with aortic or mitral insufficiency, fibroblastic endocarditis complicated by intramural thrombosis and relapsing pericarditis sometimes associated with coronary involvement are observed. Aneurysms and/or thrombosis of the coronary arteries are observed complicated by hemorragia, myocardial infarction and sudden death.

Abnormalities of the microcirculation have been described essentially through capillaroscopy (petechia, capillary dystrophy) but thet do not have any specificity.

## Articular manifestations

Arthralgia and/or arthritis occur in 45% of cases. They are frequently the presenting feature, long before the other manifestations. The knees and ankles are most involved, although smaller joints may also be affected. X-ray is generally normal. Histologically, there is an infiltration of neutrophils and mononuclear cells into the synovium and a vasculitis process on small-vessel with thrombosis. Association with HLA B27 spondylarthropathy is seen in 2% of patients. Osteonecrosis of the hip and knees have been observed but most of the patients have been treated with corticosteroids [17].

## Neurologic manifestations

They are observed in 20 to 40% of cases [18]. They may occur lately, from one to ten years after the first symptom of BD. Central nervous system involvement in BD included parenchymal and non-parenchymal (i.e. cerebral venous thrombosis or arterial aneurism) lesions [19]. Parenchymal lesions of neurobehçet’s disease frequently onset with an attack rather than a mild progressive course. They include headache, meningitis or meningoencephalitis, seizures, hemiplegia, or cranial nerve palsies. The benign intracranial hypertension is always related, in our experience of 64 cases, to cerebral venous thrombosis [20]. Psychiatric **symptoms** including **personality** changes may develop. In some cases it may be difficult to differentiate the organic manifestations from the iatrogenic side effects of therapy.

Lumbar puncture with measurement of the open pressure is mandatory. It usually shows hypercellularity; mostly lymphocytosis and less frequently polymorphonuclear cells or pleiocytosis. Cerebrospinal fluid analysis rule out an infection such as tuberculosis, listeriosis or herpes simplex virus. Typical MRI findings are small scattered lesions in multiple area of basal ganglion region, brainstem or internal capsule, with hypersignal in T2-weighted MRI and contrast enhancement with Gadolinium. They may attenuate with therapy but a retrospective diagnosis is generally feasible.

Prognosis is severe but improvement is observed with intensive and rapid therapy including corticosteroids and **immunosuppressive** drugs.

## Gastrointestinal manifestations

It is difficult to distinguish between BD and inflammatory diseases of the intestine, because of the similarity in intestinal and extra intestinal symptoms. This may explain the discrepancy of frequency ranging from 30% to 1% [21]. Gastrointestinal involvement causes nausea, abdominal pain, anorexia, diarrhea which can be bloody and **sometimes can lead toperforation.** The ileocecal region is the most commonly affected part of the gastrointestinal tract, but tranverse colon and ascending colon are sometimes involved, as is the esophagus. Histologically, the intestinal ulcers are indistinguishable from Crohn's disease, nevertheless the granuloma formation can be used to rule out BD. Cases of acute pancreatitis have been reported.

## Pulmonary manifestations

It is dominated by vascular involvement (arterial aneurysm, pulmonary embolism). Hemoptysis can be massive and fatal and is the main manifestation [14]. Pleural effusions are rare and lead to firstly rule out pulmonary embolism, tuberculosis or nosocomial infection. Cases of vasculitis have been reported.

## Genitourinary manifestations

Renal involvement is rare and dominated by AA amyloidosis occuring in patients with long standing disease not controlled with therapy or not compliant. Some cases of glomerular nephropathy mostly diffuse crescentic or focal and segmental necrotizing glomerulonephritis and IgA nephropathy have been described. Secondary effects of venous or arterial thrombosis have also been reported.

Epididymitis, occasionally recurrent, occur in 4 to 11% of patients.

## Lymphatic involvement

Lymph or splenic enlargement may exceptionally be the presenting feature and should be thoroughly evaluated. When fever is present, vascular involvement has to be searched for.

## Etiopathology

Although the pathogenesis of BD remains poorly understood, it is currently thought, as for many autoimmune or autoinflammatory syndromes, that certain infectious (in particular *Streptococcus sanguis*) and/or environmental factors are able to trigger symptomatology in individuals with particular genetic variants [22]. Streptococcal antigens were shown to increase interleukin (IL)-6 and interferon (IFN)-γ production by peripheral blood T cells from BD patients, and cross-react with a 65kD heat shock protein sharing antigenicity with oral mucosal antigens [23]. Recently, genome wide association studies from Japan and Turkey identified an association at IL23R and IL12RB2 locus [24,25]. The implication of T cells and polymorphonuclear leukocytes is supported by pathological studies showing perivascular infiltration of memory T cells and polymorphonuclear leucocytes within vasculitic lesions in BD patients with arterial and central nervous system (CNS) involvement [26,27]. However, the nature of T cells driving inflammatory lesions remains elusive. We recently demonstrated the promotion of Th17 responses and the suppression of regulatory T cells (Tregs) that were induced by interleukin (IL)-21 production and that correlates with BD activity [28]. We demonstrated the presence of IL-21 and IL-17A-producing T cells within cerebrospinal fluid (CSF), brain parenchyma inflammatory infiltrates, and intracerebral blood vessels from active BD patients with central nervous system (CNS) involvement. Stimulation of CD4^+^ T cells with IL-21 increased Th17 and Th1 differentiation and decreased Tregs frequency. Conversely, IL-21 blockade with an IL-21R-Fc restored the Th17 and Treg homeostasis in BD patients. Our findings suggest that IL-21 exerts a critical role in the pathogenesis of BD, thus providing a promising target for novel therapy [28].

## Diagnosis

In BD there is no relevant biological test for diagnosis. International criteria of classification **have** been defined with a sensitivity of 85% and specificity of 96% [3]. The erythrocyte sedimentation rate, CRP and other acute phase reactants are seldom elevated during the acute phase and/or relapses of BD but are not well correlated with disease activity. Abnormalities of fibrinolysis, elevated factor VIII, immune circulating complexes and cryoglobulinemia have been occasionally reported. Leucocytosis is frequently encountered. The positivity of HLA B 51 allele is of no diagnostic value. Cutaneous biopsy of intradermal injection with physiologic saline solution may demonstrate vasculitis with immune complexes deposit.

## Differential diagnosis

The diagnosis of BD is only supported by clinical criteria that require the exclusion of other diagnoses based on clinical presentation. Oral ulceration is not specific of BD as it may occur in 30-40% of the general population. In contrast, bipolar ulcerations are more specific of BD. Oral ulcerations may also be associated with hemopathy, HIV, Crohn’s disease, lupus, bullous dermatosis or vitamin deficiencies. Sarcoidosis, Crohn’s disease, Vogt-Koyanagi Harada and infectious uveitis must be ruled out in case of recurrent uveitis.

Venous involvement should exclude the antiphospholipid syndrome, or thrombophilia. Arterial lesions of BD may mimic Takayasu’s arteritis or polychondritis. Neuro-BD is sometimes difficult to distinguish from multiple sclerosis or tuberculosis. Lastly, chronic inflammatory bowel disorders must be ruled out in case of gastrointestinal involvement.

## Management

Due to the lack of an etiologic agent, the treatment is symptomatic without consensus. EULAR recommendations for the management of BD, based on the available literature and expert opinions, have been recently proposed [29]. The goals are the functional recovery of a visceral involvement (eye, CNS) and prevention of relapse(s). The risks of BD are an **increased** mortality especially in case of arterial involvement, and a high morbidity due to the cumulative sequellae of ocular and neurological involvement [7,30]. Steroids are the corner stone of the antiinflammatory agents administered topically (anterior uveitis) or systemically. A general consensus is obtained for their prescription in case of ocular and/or central nervous system involvement at a dosage of 1mg/kg/day. Initiation of therapy could be made by infusion of methylprednisolone (1 g.) during the first three days. When steroids are used, they can be reduced with caution after 4 weeks. Relapses are frequently seen after discontinuation of steroids. Corticodependance is frequently observed. Immunosuppressive drugs have been shown to be effective. Due to their delay of action, they are prescribed initially in association with corticosteroids. They are prescribed in cases of severe organ involvement (i.e. posterior uveitis, CNS involvement, vascular involvement…). Azathioprine (2.5 mg/kg/day) was proved effective in a controlled study [31]; cyclophosphamide orally (2 mg/kg/day) or intravenously (750 to 1g/m^2^ every 4 weeks) is also used. The efficacy of oral methotrexate (7.5 mg once a week) has also been reported. Chloraminophen (0.1 to 0.2 mg/kg/day) is less presribed due to its hematological side effects. Ciclosporin was proved effective in uveitis [29], but secondary nephropathy limits its prescription. More recently the efficacy of infliximab has been reported in severe cases of BD uveitis [32]. Plasmapheresis and intravenous immunoglobulins were efficient in anecdotal reports. Alpha interferon (2a or 2b) is also efficient, especially in case of severe and/or resistant uveitis [33].

We usually prescribe antiagregant therapy or anticoagulation in case of vascular involvement.

Colchicine (1-2mg/day) has beneficial effects on the mucocutaneous symptoms decreasing the number, size and recurrence of aphthae [34]. We used it systematically in adjunction with other therapeutic agents. Severe flares of BD were observed after cessation of this drug.

Thalidomide is also reported to be effective for oral and genital ulcers and pseudofolliculitis with relapses when stopped [35]. Contraceptive measures are mandatory due to severe fœtal malformation. Among side effects, peripheral neuropathy is frequent.

Disulone, Sucralfate, and **Pentoxifylline have shown some efficacy in anecdotal** reports [36,37].

As for other chronic diseases, patient education, good compliance, regular follow-up and rapid therapeutic administration improve the prognosis.

## Prognosis

BD significantly increases morbidity and mortality. The leading causes of morbidity in BD are the uveitis with the potential threat of visual loss and neurologic involvement. Few, studies have addressed the mortality of Behçet syndrome. Among 2,031 patients from Japan, 31.7% were clinically deteriorated, and 0.9% died during the course of a single year’s follow-up. In Turkey, 42 patients out of 428 died mainly due to major vessel disease and neurologic involvement. We recently reported that among 817 BD patients, 41 (5%) died after a median follow-up of 7.7 years [7]. The mean (± SD) age at death was 34.6 ± 11.5 years with 95.1% of male. Main causes of death included major vessel disease (mainly arterial aneurysm and Budd-Chiari syndrome) (43.9%), cancer and malignant hemopathy (14.6%), and central nervous system involvement and sepsis (12.2%). The mortality rate at 1 and 5 years was of 1.2% and 3.3%, respectively. There was an increased mortality among the 15–24 years [SMR with 95% confidence interval, 2.99 (1.54-5.39)], and the 25–34 years, [SMR 2.90 (1.80-4.49)] as compared to age and sex matched healthy controls. The mortality decreased in patients older than 35 years [SMR, 1.23 (0.75-1.92)]. In multivariate analysis, male gender (HR: 4.94, CI: 1.53-16.43), arterial involvement (HR: 2.51, CI: 1.07-5.90), and a high number of BD flare (HR: 2.37, CI: 1.09-5.14) were independently associated with mortality [7].

## Competing interests

The authors declare that they have nothing to disclose.

## Authors’ contributions

ADS and BW wrote the manuscript. DS and BW read and approved the final manuscript.
